# Quantitative Imaging Biomarkers of PRP-Induced Tendon Remodelling in Chronic Tendinopathy: Review and Single-Centre Experience with Ultrasound Radiomics and MRI T2 Profiling

**DOI:** 10.3390/diagnostics16081233

**Published:** 2026-04-20

**Authors:** Živa Miriam Geršak, Karlo Pintarić, Jernej Vidmar, Vladka Salapura

**Affiliations:** 1 Clinical Institute of Radiology, University Medical Centre Ljubljana, 1000 Ljubljana, Slovenia; ziva.gersak@kclj.si (Ž.M.G.); vladka.salapura@kclj.si (V.S.); 2 Department of Radiology, Faculty of Medicine, University of Ljubljana, 1000 Ljubljana, Slovenia; karlo.pintaric@kclj.si; 3 Institute of Physiology, Faculty of Medicine, University of Ljubljana, 1000 Ljubljana, Slovenia

**Keywords:** quantitative imaging biomarkers, chronic tendinopathy, platelet-rich plasma, US radiomics, T2 mapping

## Abstract

Platelet-rich plasma (PRP) is widely used as a second-line treatment for chronic tendinopathy that persists despite structured conservative care, yet outcomes and imaging correlates remain heterogeneous. This review outlines PRP biology and preparation, summarises quantitative imaging techniques for monitoring tendon response, and presents the experience of a single centre integrating these methods into routine supraspinatus and lateral elbow PRP workflows. PRP is described as an autologous platelet concentrate with variable leukocyte and fibrin content, with leukocyte-rich formulations commonly selected for chronic tendinopathy. Quantitative approaches—including ultrasound shear-wave elastography and radiomics, MRI T2/T2* mapping, CT-based bone metrics, PET/CT, and optical techniques—offer numerical biomarkers of tendon structure, mechanics, and inflammation but are rarely implemented in PRP trials. At the authors’ centre, leukocyte-rich PRP is injected under ultrasound guidance after failed physiotherapy, and follow-up combines validated questionnaires with grey-level run-length matrix texture analysis of ultrasound and 3.0 T MRI T2 distribution profiling. A pilot ultrasound study in supraspinatus and common extensor tendinosis showed uniform short-term clinical improvement and significant changes in most texture features, with selected parameters correlating with symptom relief. A prospective supraspinatus cohort demonstrated significant six-month clinical gains in both tendinosis and small partial-thickness tears, whereas only the tendinosis group exhibited T2 profile convergence toward asymptomatic patterns. These data indicate that quantitative ultrasound radiomics and whole-length T2 profiling are feasible imaging biomarkers that capture PRP-induced tendon remodelling beyond qualitative imaging and may help tailor PRP protocols to specific tendon phenotypes.

## 1. Review

### 1.1. Introduction

Degenerative tendon disorders are among the most frequent and disabling musculoskeletal conditions, causing pain, functional limitation, and reduced quality of life in both athletic and general populations. Rotator cuff, patellar, Achilles, and lateral elbow tendinopathies are particularly prevalent and often long-standing, frequently persisting for many months despite guideline-based conservative care and contributing substantially to work absenteeism, reduced productivity, healthcare use, and career-limiting disability in high-performance athletes [1,2,3,4,5].

Conservative management centred on education, load modification, and exercise therapy is the recommended first-line strategy, but fails to resolve symptoms in many patients with chronic tendinopathy fully. Platelet-rich plasma (PRP) has therefore become a widely used second-line option that delivers a concentrated mixture of platelets and growth factors to modulate inflammation and promote tendon repair. Systematic reviews and meta-analyses indicate that, particularly after failed structured conservative treatment, PRP provides modest yet clinically relevant long-term pain reduction in chronic tendinopathy. In contrast, functional gains are less consistent, and trial heterogeneity in patient selection, PRP formulation, and control interventions remains considerable. In rotator cuff and patellar tendinopathy, randomised trials and pooled analyses since 2020 report that PRP can improve pain and some functional outcomes, but effect size and durability depend on tendon location, leukocyte content and dosing of PRP, and whether comparators consist of saline, corticosteroid, or rehabilitation alone [2,6,7,8,9,10].

Outcome assessment in tendinopathy is likewise moving from purely qualitative imaging toward quantitative evaluation of tendon structure and biomechanics. Conventional ultrasound and MRI remain indispensable for diagnosis and grading, but are largely qualitative or semi-quantitative and show limited sensitivity to subtle remodelling after biologic therapies such as PRP. Advanced methods—including shear-wave elastography, ultrasound tissue characterisation, MRI T2/T2* and T1ρ mapping, ultrashort-echo-time and diffusion-based sequences, perfusion imaging, and radiomics-based texture analysis—provide numerical biomarkers of tendon composition, stiffness, and microstructure. These techniques have been increasingly applied to tendon research. However, their use in PRP studies is still sparse, acquisition and analysis protocols are not standardised, and relationships between quantitative imaging changes and patient-reported outcomes remain incompletely defined [3,9,11,12,13].

These knowledge gaps justify both a synthesis of available data on quantitative assessment of tendon response after PRP and a detailed description of real-world implementations of such methods. Our medical centre has developed and applied quantitative ultrasound texture analysis and MRI-based mapping to monitor tendon remodelling after PRP injections in supraspinatus and other tendinopathies, providing practical examples of how these techniques can complement clinical evaluation [5,6,11,14,15].

The main aim of this review is to summarise the current basic principles of PRP treatment in tendinopathy, outline existing quantitative methods for assessing tendon changes after PRP injections, and contextualise the quantitative imaging experience of our medical centre within the contemporary literature. Within this framework, the single-centre clinical cohorts are presented not as stand-alone trials, but as pragmatic case studies based on two previously published studies that are summarised here to illustrate how ultrasound radiomics and MRI T2 profiling can be implemented in routine PRP workflows and how their quantitative readouts map onto patient-reported outcomes.

### 1.2. PRP Versions

Platelet-rich plasma (PRP) is an autologous blood product produced by centrifuging anticoagulated whole blood to obtain plasma with platelet levels typically two- to five-fold above baseline. Alongside platelets carrying growth factors (e.g., PDGF, TGF-β, VEGF, IGF, FGF), PRP contains variable numbers of leukocytes, residual erythrocytes, and plasma proteins, including fibrinogen, fibronectin, and vitronectin. After activation, this cargo can modulate inflammation, promote the chemotaxis and proliferation of reparative cells, enhance angiogenesis, and support matrix synthesis, supporting its use in degenerative tendon and other musculoskeletal disorders [16,17,18,19,20].

PRP is prepared by drawing venous blood into citrate tubes and processing it in a centrifuge or a closed system. Centrifugation separates red cells, the platelet/leukocyte-rich buffy coat, and platelet-containing plasma. Single-spin protocols are fast but yield moderate platelet enrichment with substantial leukocyte retention. In contrast, double-spin protocols add a second, higher-speed spin to pellet platelets, discard platelet-poor plasma, and resuspend the pellet in a smaller volume, increasing platelet concentration and enabling partial control of leukocyte content [16,17,21,22,23].

Platelet enrichment depends on starting volume, centrifugal force and time, and kit geometry, with many protocols targeting a roughly three- to five-fold increase over baseline. Low enrichment may reduce efficacy, while very high concentrations may inhibit cell proliferation and potentially impair repair. PRP may be injected non-activated (relying on in vivo activation) or activated ex vivo with calcium chloride or thrombin to form a fibrin gel and trigger growth-factor release [16,17,21,22,23,24].

Because preparations vary in platelet dose, leukocyte/red-cell content, fibrin structure, and activation, classification systems aim to improve comparability and guided use. A widely used framework separates four families: pure platelet-rich plasma (P-PRP) and leukocyte- and platelet-rich plasma (L-PRP) as liquids, and pure platelet-rich fibrin (P-PRF) and leukocyte- and platelet-rich fibrin (L-PRF) as solid fibrin clots. Clinically, P-PRP is leukocyte-poor and used when a lower inflammatory profile is desired. In contrast, L-PRP is leukocyte-rich and used to induce a stronger inflammatory/remodelling response, often with more post-injection soreness [16,21,25,26,27].

P-PRF and L-PRF are prepared without anticoagulant, forming clots or membranes that entrap platelets (and, in L-PRF, leukocytes) and release growth factors gradually over days; they are mainly used in oral/maxillofacial and some orthopaedic surgery rather than as injectables. More detailed schemes (PAW, DEPA, MARSPILL) also describe platelet concentration/dose, activation method, white- and red-cell content, and production efficiency, and guidelines recommend reporting at least platelet counts, leukocyte and red-cell content, activation status, and whether the product is plasma- or fibrin-based [16,22,25,26,28].

In musculoskeletal and tendinopathy-focused practice, these taxonomies are often simplified to leukocyte-poor PRP (typically P PRP) versus leukocyte-rich PRP (L PRP), with selection based on the desired biological profile. Leukocyte-poor PRP is commonly preferred intra-articularly or where limiting additional inflammation and catabolic activity is important. In contrast, leukocyte-rich formulations are used in some chronic tendinopathy/enthesopathy protocols to drive a stronger inflammatory and remodelling response, which is important when interpreting heterogeneous clinical and imaging outcomes [16,17,22,27].

### 1.3. Quantitative Methods of Assessment

Quantitative assessment methods in tendinopathy are designed to move beyond qualitative impressions of tendon “thickening” or “heterogeneity” and instead provide numerical biomarkers of structure, composition, and biomechanics that can be tracked over time. These techniques include quantitative MRI mapping, ultrasound-based methods such as elastography and texture analysis, and, in more specialised settings, PET/CT and optical approaches, each probing different aspects of tendon pathology and responses to therapies such as PRP. By reducing observer dependence and detecting subtle, spatially distributed changes, quantitative methods are particularly suited to monitoring biologic treatments that induce gradual remodelling rather than gross anatomical alterations [13,29,30,31,32].

#### 1.3.1. Ultrasound Radiomics and Elastography

In this review, we use the term ‘texture analysis’ to refer specifically to second- and higher-order greyscale statistics (such as GLCM and GLRLM features) extracted from ultrasound images, whereas ‘radiomics’ denotes the broader framework of quantitative feature extraction that includes texture features alongside other numerical descriptors.

Ultrasound is the most accessible tendon imaging modality, and advanced techniques such as shear-wave elastography and texture analysis provide quantitative measures of tendon stiffness and echotexture. Shear-wave elastography quantifies shear-wave velocity or elastic modulus, can distinguish tendinopathic from normal tendons, and yields stiffness values that correlate with pain and functional limitation. These elastography metrics are highly sensitive to probe pressure, region-of-interest placement, and tendon anisotropy, necessitating strict standardisation of acquisition protocols in longitudinal PRP studies. Ultrasound texture analysis derives higher-order statistical features, including grey-level co-occurrence (GLCM) and run-length (GLRLM) metrics, that describe the spatial distribution of pixel intensities within the tendon. Systematic data indicate that musculoskeletal ultrasound texture analysis is feasible and sensitive to changes in muscle quality and tendon pathology. Still, the evidence base is constrained by small, heterogeneous cohorts and limited correlation with clinical outcomes [30,31,33,34].

Radiomics in musculoskeletal ultrasound refers to extracting large numbers of quantitative features from B-mode images that describe greyscale intensity and its spatial arrangement within a predefined region of interest. This approach extends conventional measures such as tendon thickness or mean echogenicity into a multidimensional set of numerical descriptors of tendon structure. Radiomic features are typically grouped into first-order histogram metrics that summarise intensity distributions, second-order grey-level co-occurrence matrix features that capture spatial relationships between pixel pairs, and run-length or other higher-order features that characterise the length and uniformity of contiguous grey-level runs [32].

Tendon echotexture reflects collagen fibre orientation, packing density, hydration, focal degeneration, neovascularisation, and interfaces with adjacent tissues. Radiomic features that capture streak length and grey-level uniformity, such as GLCM contrast and GLRLM short-run and long-run emphases, provide quantitative indices of fibre organisation and microstructural disruption that are not discernible on qualitative imaging. In musculoskeletal ultrasound, radiomics has been applied mainly to skeletal muscle to distinguish neuromuscular disease patterns, grade frailty and sarcopenia, and monitor adaptations to exercise or rehabilitation. In contrast, applications of tendon and PRP treatments for tendinopathy remain limited [32].

Robust tendon radiomics requires standardised B-mode acquisition with fixed probe type, frequency, depth, gain, focus, and patient positioning. Longitudinal regions of interest should be reproducibly placed over the pathological tendon segment while excluding bone, bursa, and marked anisotropy. A recent systematic review found that musculoskeletal ultrasound texture analysis is technically feasible and clinically promising. Still, it emphasised small, heterogeneous samples, non-uniform feature implementation, and infrequent linkage to clinical endpoints as major barriers to broader adoption [32].

#### 1.3.2. MRI T2/T2* Mapping and Relaxation Profiling

Quantitative MRI T2 and T2* mapping provides voxel-wise relaxation times that reflect collagen organisation, water content, and matrix integrity in tendon tissue. In healthy rotator cuff tendons, densely packed, highly ordered collagen with minimal free water produces very short T2 values, whereas degeneration, micro-tearing, mucoid change, and interstitial oedema prolong T2 and T2*. Multi-echo sequences are acquired, and the transverse magnetisation decay is fitted pixel-by-pixel to generate colour-coded T2 or T2* maps. Relaxation times are sampled within predefined regions of interest or along the tendon to quantify focal and regional pathology [13,14,30].

Cross-sectional supraspinatus studies at 1.5 T show that T2 and T2* mapping can distinguish asymptomatic tendons from tendinosis and partial- or full-thickness tears, with the highest accuracy when the lateral tendon segment is sampled in both coronal and sagittal planes. These studies report excellent intra- and inter-observer reproducibility and support lateral-segment cutoff values for grading pathology severity. Histogram-, texture-, and cluster-based analyses of T2 maps further quantify tissue heterogeneity and may more closely correspond to the clinical spectrum of pain and functional deficit [7,13,14,35,36].

In longitudinal monitoring of biologic therapies, analysing the spatial profile of T2 values along the tendon provides additional information beyond static mean segmental values. Changes in profile slope, intercept, or contour can indicate redistribution of relaxation times consistent with partial normalisation of matrix composition and collagen architecture, even when segmental mean T2 remains unchanged. Integrating tendon-specific measures, such as supraspinatus T2 profiling, with quantitative cartilage and muscle mapping may yield a more comprehensive assessment of shoulder joint status in patients undergoing PRP or other orthobiologic interventions [14,29,37].

#### 1.3.3. Other Quantitative Methods

PET/CT provides complementary imaging of inflammatory and metabolic activity around tendons and entheses. 18F-FDG PET/CT studies suggest standardised uptake values can reflect inflammatory burden and track treatment response in systemic inflammatory disease, supporting the concept of quantitative musculoskeletal “inflammatory load” imaging. However, radiation exposure, cost, and limited spatial resolution currently confine PET/CT to selected scenarios rather than routine follow-up of degenerative tendinopathy [35,36,38,39,40,41,42].

Optical methods, such as polarisation-sensitive optical coherence tomography (PS-OCT), quantify fibrous structure by measuring birefringence as a surrogate for collagen alignment. In experimental tendon injury models, PS-OCT metrics track healing and correlate with histology, but clinical tendon use remains limited to surgically accessible or research settings [43,44].

## 2. Our Centre’s Experience

In this section, we do not aim to provide definitive efficacy data, but rather to illustrate, through two pragmatic pilot cohorts, how quantitative ultrasound radiomics and MRI T2 profiling can be embedded in everyday PRP practice. These real-world examples are intended to complement the literature review by providing concrete imaging workflows, feature extraction strategies, and early signals linking quantitative biomarkers to symptom change.

At our centre, PRP is offered as a minimally invasive treatment for chronic tendinopathy in patients with symptoms persisting for at least three months despite at least one completed cycle of physiotherapy, with a predominant focus on supraspinatus tendinopathy and lateral epicondylitis. PRP is prepared as a leukocyte-rich concentrate, administered under ultrasound guidance after standardised clinical and imaging evaluation, and treatment response is monitored using validated questionnaires complemented by quantitative ultrasound or MRI mapping chosen to detect subtle tendon remodelling [14,15].

At our centre, PRP is prepared as leukocyte-rich plasma using a double-spin protocol, as described in our previous publications. Mean platelet concentration in the applied PRP is approximately 3–5-fold above baseline (around 1.9 × 10^6^ platelets/µL), with leukocyte counts of roughly 20–25 × 10^3^/µL and some neutrophil content. PRP is injected non-activated under ultrasound guidance as a single injection per affected tendon, and repeat injections are not routinely scheduled in these pilot protocols.

**Figure 1:**
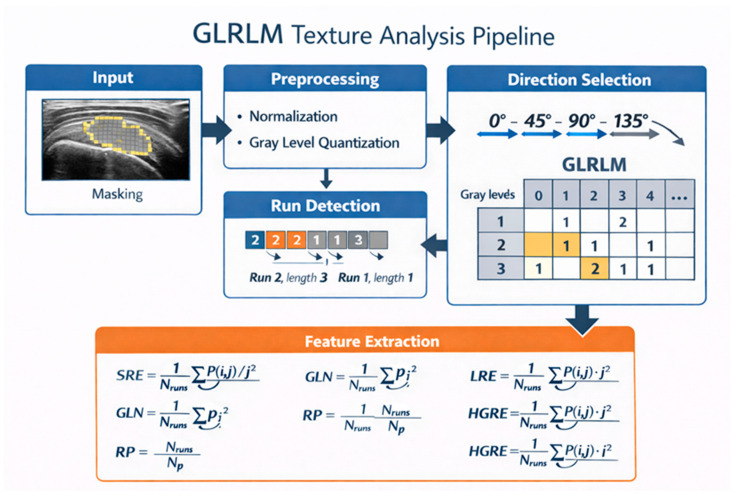
Longitudinal ultrasound image of supraspinatus tendinosis with ROI from the tendon footprint to the myotendinous junction. Additional panels show the GLRLM texture analysis pipeline.

In the first pilot study, thirteen patients with symptomatic tendinosis of either the supraspinatus tendon (n = 4) or the common extensor tendon of the forearm (n = 9) received a single ultrasound-guided RP injection. They were reassessed after three months, with SPADI used for supraspinatus tendinopathy and PRTEE for lateral epicondylitis. All participants reported at least some improvement in pain and disability scores over this interval. Standardised longitudinal grey-scale ultrasound images of the most affected tendon segment were acquired before and after PRP and analysed using GLRLM-based texture features, as shown in Figure 1 [14,15].

In our published pilot study on supraspinatus and common extensor tendinosis, ultrasound radiomics was performed using the following standardised pipeline. Texture analysis (Figure 1) was performed offline on anonymised 8-bit DICOM images using ImageJ v1.54 (National Institutes of Health, Bethesda, Maryland, United States) for image handling and R (version 4.1.2, Radiomics Image Processing Toolbox v0.1.3) for GLRLM feature extraction. For each examination, one longitudinal B-mode image with maximal tendinopathic involvement was selected using a standardised preset (fixed probe, frequency, depth, gain, focus). Images were resampled to an in-plane pixel size of approximately 0.1 mm × 0.1 mm, converted to 8-bit, and grey levels were discretised into 64 bins. A rectangular region of interest was drawn along the most affected tendon segment, excluding cortical bone, bursa and shadowing. The same sonographer reproduced ROI placement at follow-up using anatomical landmarks and stored probe-position screenshots. Grey-level run-length matrices were computed in the longitudinal fibre direction (0°), with run length up to the full ROI length. From each GLRLM, standard features were extracted, including short-run emphasis, long-run emphasis, low-grey-level run emphasis, high-grey-level run emphasis and long-run low-grey-level emphasis, following IBSI definitions [14,15]. In our own pilot cohorts, we did not use shear-wave elastography; all quantitative ultrasound analyses were based on B-mode images and GLRLM-derived radiomics features rather than elastography-derived stiffness metrics.

Significant changes were observed in nearly all GLRLM parameters, and long-run low-grey-level emphasis and low-grey-level run emphasis showed statistically significant correlations with clinical improvement. In practical terms, patients who reported the largest reductions in pain and disability tended to show a relative loss of long hypoechoic runs and a shift toward shorter, more homogeneous grey-level streaks, consistent with partial restoration of the organised fibrillar pattern. This suggests that GLRLM-derived metrics capture microstructural remodelling that parallels symptomatic relief [14,15].

In the study, GLRLM replaced the semi-quantitative evaluation of tendinopathy based on “classic” US features (loss of longitudinal fibrillar pattern, heterogeneous texture) and enabled quantification of tendon properties from their texture content, thereby enabling a more objective analysis of tissue imaging and clinical outcomes after PRP. However, despite the method’s objective nature, it still depends on the operator’s experience and technical precision in handling the ultrasound probe, acquiring images, and performing the measurements required for quantitative assessment of tissue changes. This operator dependence also affects longitudinal studies, in which different examiners may be involved over time. A potential solution is the fusion of ultrasound with MRI to enable more precise and reproducible selection of the region of interest [15].

**Figure 2:**
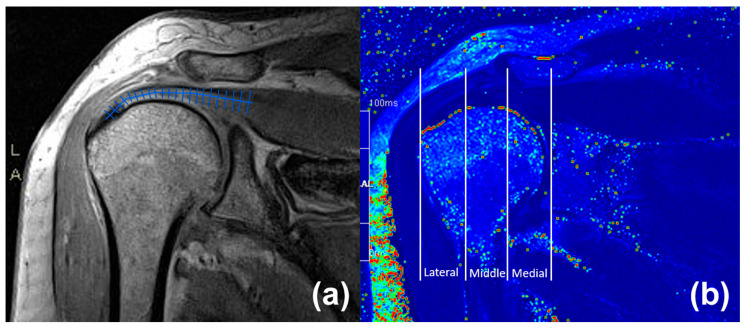
(**a**) Coronal T2 map and schematic showing division of the supraspinatus tendon into lateral, middle, and medial segments, with T2 profiling along its length. (**b**) Manual segmentation from the tendon footprint to the myotendinous junction for T2 analysis.

In the second study, a monocentric prospective cohort of twenty-six patients with supraspinatus tendinopathy was treated with a single PRP injection and stratified according to baseline MRI into a tendinosis group (n = 9) and a partial-thickness tear group involving less than 50% of tendon thickness (n = 13), with an additional four asymptomatic volunteers serving as controls for quantitative T2 analysis. All patients underwent 3.0 T MRI with a dedicated T2-mapping sequence and were clinically assessed with the SPADI and Constant–Murley scores at baseline, at 1 month (SPADI), and at 6 months (both scores) [14]. Quantitative MRI was performed on a 3.0 T scanner using a dedicated multi-echo spin-echo (for T2), with a shoulder coil and standard clinical positioning. Echo times, repetition time, slice thickness and field of view were kept constant across all examinations. For each subject, the supraspinatus tendon was manually segmented on the T2 maps from the footprint to the myotendinous junction on coronal images, creating a full-length tendon mask. The tendon was then divided into lateral, middle and medial thirds to derive segmental T2 values. In addition, T2 distribution profiling was performed by sampling relaxation times along the normalised tendon length to obtain continuous profiles (Figure 2). Maps with motion or fitting artefacts were excluded. All segmental values and profile metrics were exported for statistical analysis [14].

Both structural groups showed significant clinical improvement over 6 months. The structure of the supraspinatus tendon was evaluated using quantitative T2 mapping with full-tendon segmentation and T2 distribution profiling, extending prior work that predominantly relied on small ROI-based measurements. By creating masks from the tendon footprint to the myotendinous junction and averaging all voxels within three anatomical segments, the study confirmed the characteristic lateral-to-medial increase in T2 in healthy tendons. It demonstrated that tendinosis and small partial tears show consistently higher T2 values across all segments, consistent with collagen disorganisation, fibre disruption, increased mucoid matrix, and elevated water content. Compared with earlier 1.5 T protocols that sampled limited areas and frequently grouped partial- and full-thickness tears, this approach offers higher signal sensitivity and a more exhaustive representation of tendon tissue, while acknowledging some loss of focal detail through averaging. To capture spatial heterogeneity more precisely, T2 distribution profiling was additionally applied to derive continuous T2 curves along the normalised tendon length. This method revealed abnormal biphasic profiles with altered lateral slopes in diseased tendons and uncovered PRP-induced remodelling in tendinosis—manifested as a more “healthy-like” lateral slope and reduced offset—that was not evident from segmental mean T2 values alone. The key strengths of this quantitative framework are its sensitivity to regional structural changes, its capacity to differentiate tendinosis and partial tears from asymptomatic tendons, and its potential as an imaging biomarker of response to biologic therapy. Nonetheless, widespread implementation is constrained by the need for dedicated sequences, sophisticated post-processing, and standardised protocols, as well as by the limitations of a small pilot cohort with heterogeneous, non-stratified partial tears and a 6-month follow-up, which restricts subgroup analyses. Future studies should focus on automating segmentation and profile generation, incorporating detailed tear morphology, and validating these techniques in larger, multicenter populations with extended follow-up to enable broader clinical use of T2 mapping and T2 distribution profiling in tendon imaging [14]. From a biological perspective, the observed ultrasound and MRI changes are consistent with early matrix remodelling in the tendinopathic tendon. Quantitative texture shifts away from long hypoechoic runs toward shorter, more homogeneous streaks, together with T2 profile convergence toward control-like values, likely reflect improved collagen fibre organisation, reduced interfibrillar water, and partial normalisation of proteoglycan-rich mucoid matrix. These imaging signatures support the concept that PRP primarily modulates collagen architecture and tissue composition in tendinosis, rather than simply altering gross morphology [14]. To improve the clarity and translational value of our single-centre experience, we additionally provide a schematic overview of the clinical PRP workflow integrating quantitative imaging endpoints (Table 1). The flowchart summarises patient selection following failed conservative treatment, standardised preparation and ultrasound-guided administration of leukocyte-rich PRP, and structured follow-up combining clinical outcome measures with quantitative imaging. In this framework, ultrasound radiomics is applied at short-term follow-up to capture early microstructural changes, whereas MRI T2 mapping and distribution profiling are used at mid-term follow-up to assess spatial patterns of tendon remodelling. This integrated approach highlights how quantitative imaging biomarkers can be embedded into routine clinical pathways and aligned with symptom evolution.

Together, these two studies show that integrating ultrasound texture analysis and MRI T2 distribution profiling into PRP protocols at our centre is feasible, and that short-term GLRLM-based echotexture changes and medium-term T2 profile normalisation, predominantly in tendinosis, provide complementary information to clinical scores. These techniques emerge as candidate imaging biomarkers for tendon response and as tools for planning larger PRP trials with quantitative imaging endpoints [14,15].

Post-treatment monitoring combines clinical outcome measures with quantitative imaging at predefined time points. Short-term follow-up (approximately 3 months) includes ultrasound-based radiomics analysis using grey-level run-length matrix (GLRLM) features, while mid-term follow-up (approximately 6 months) incorporates quantitative MRI with T2 mapping and T2 distribution profiling. Clinical and imaging data are subsequently integrated to evaluate PRP-induced tendon remodelling and its relationship to symptom improvement.

## 3. Comparison with Other Similar Studies

In this section, we position our single-centre quantitative imaging experience within the broader PRP and imaging biomarker literature summarised above. In the ultrasound domain, our GLRLM-based texture-analysis study demonstrated significant three-month changes in most texture features after PRP for supraspinatus and common extensor tendinosis, and identified long-run low-grey-level emphasis and low-grey-level run emphasis as parameters correlating with symptomatic improvement. Most other quantitative ultrasound studies in PRP-treated tendinopathy have focused on shear-wave elastography, in which PRP has been associated with increased tendon stiffness and improved clinical scores in conditions such as lateral epicondylitis and Achilles tendinopathy. Still, these trials have typically relied on single stiffness metrics without detailed textural characterisation. This contrast suggests that higher-order texture features provide information about spatial echotexture organisation that is not captured by morphology or stiffness alone and may be particularly sensitive to early PRP-induced changes, positioning ultrasound radiomics as a logical next step beyond elastography in quantitative tendon assessment [14,32,34,37,45].

With respect to MRI, our supraspinatus study found that mean segmental T2 values remained largely unchanged after PRP, whereas T2 distribution profiles revealed segment-specific remodelling and convergence toward control profiles in tendinosis but not in partial-thickness tears. This longitudinal pattern extends earlier quantitative MRI work that used single-time-point T2/T2* mapping to differentiate supraspinatus tendinosis and tears from asymptomatic tendons and complements PRP trials that relied on conventional MRI or MR arthrography to assess tear size and signal intensity, where structural changes have been inconsistent and often poorly correlated with clinical outcomes. The observation that both tendinosis and partial-tear groups show robust clinical improvement, but clear quantitative MRI normalisation occurs only in tendinosis, is consistent with experimental data indicating that biologic therapies more readily reverse matrix-level degeneration than repair structural discontinuities and underscores the added value of whole-length T2 profiling over simple mean T2 or qualitative grading when monitoring treatment response [14,46,47,48].

Systematic reviews and meta-analyses on PRP for rotator cuff and other tendinopathies consistently describe modest long-term pain relief, variable functional benefit, and substantial heterogeneity in PRP formulation, dosing, and outcome measures, while noting that imaging follow-up is usually qualitative and rarely employs advanced quantitative techniques. Within this context, our centre’s experience demonstrates that ultrasound texture analysis and quantitative MRI can be embedded in routine PRP workflows for upper-limb tendons and offers early evidence that these biomarkers distinguish tissue-level responses between tendinosis and partial-thickness tears even when clinical improvement appears similar. These findings support the inclusion of standardised radiomics and mapping protocols in future PRP trials to refine patient selection, optimise PRP strategies, and better understand why certain tendon phenotypes display more pronounced structural remodelling than others [5,7,15,32,49].

## 4. Conclusions

Platelet-rich plasma has become an established second-line option for chronic tendinopathy that is unresponsive to structured conservative care, yet clinical outcomes and imaging correlates remain heterogeneous across tendons, PRP preparations, and study designs. Quantitative imaging techniques, including ultrasound radiomics and MRI T2/T2* mapping, offer sensitive numerical descriptors of tendon structure and composition that may capture subtle remodelling beyond the resolution of conventional qualitative assessment.

In this narrative review, we summarised key principles of PRP preparation and application in tendinopathy, outlined the current evidence on quantitative tendon imaging, and illustrated, through two previously published single-centre pilot cohorts, how ultrasound GLRLM-based texture analysis and whole-length T2 distribution profiling can be integrated into routine PRP workflows. These cohorts demonstrate the feasibility of implementing standardised acquisition, segmentation, and feature-extraction pipelines in everyday practice and suggest that selected radiomics and T2 profiling metrics are sensitive to PRP-associated remodelling signals, particularly in tendinosis phenotypes.

However, both cohorts are small, uncontrolled, single-arm series without sham, saline, or physiotherapy comparators, lack a priori power calculations, and do not include formal reproducibility studies or multiple-comparison adjustments, so the findings are inherently exploratory and hypothesis-generating. Importantly, the available data do not yet demonstrate clear clinical utility, predictive capability, or a direct influence on treatment decision-making; at present, these quantitative biomarkers should therefore be regarded primarily as research tools for phenotyping tendon pathology and monitoring biological response.

Future work should focus on multicentre, adequately powered randomised trials that embed standardised quantitative imaging protocols, pre-specified responder definitions, and imaging-guided decision algorithms to test whether ultrasound radiomics and T2 profiling can move from feasibility signals to validated adjuncts for patient stratification, prognostication, and tailoring of PRP or other orthobiologic interventions in chronic tendinopathy.

## Figures and Tables

**Table 1:** Clinical PRP workflow integrating quantitative imaging endpoints. The flowchart illustrates the standardised workflow implemented at our centre for the management of chronic tendinopathy using platelet-rich plasma (PRP). Patients with persistent symptoms despite prior conservative treatment undergo baseline clinical and imaging assessment. Following selection, leukocyte-rich PRP is prepared and administered under ultrasound guidance.

Step	Clinical Stage	Procedures/Actions	Imaging Endpoints	Outcome Measures
1	Patient selection	Chronic tendinopathy with persistent symptoms ≥3–6 months despite structured conservative treatment	Baseline ultrasound and/or MRI (qualitative assessment)	Baseline clinical scores (SPADI, PRTEE, Constant–Murley)
2	Baseline evaluation	Standardised clinical and imaging assessment	Optional baseline quantitative imaging (if available)	Symptom severity, functional limitation
3	PRP preparation	Single-spin protocol; leukocyte-rich PRP (L-PRP); ~3–5× platelet concentration	—	—
4	PRP administration	Ultrasound-guided injection; single, non-activated PRP injection	Real-time ultrasound guidance	Procedural accuracy
5	Short-term follow-up (~3 months)	Clinical reassessment	Ultrasound radiomics (GLRLM texture analysis)	Changes in pain and function; correlation with texture features
6	Mid-term follow-up (~6 months)	Clinical reassessment	MRI T2 mapping and T2 distribution profiling	Structural tendon changes; T2 profile normalisation
7	Data integration	Combined analysis	Integration of US radiomics and MRI biomarkers	Correlation between imaging biomarkers and clinical improvement
8	Interpretation	Treatment response evaluation	Quantitative assessment of tendon remodelling	Identification of responders and phenotype-specific effects

## Data Availability

The data that support the findings of this study are available from the corresponding authors upon reasonable request.

## Abbreviations

The following abbreviations are used in this manuscript:

3.0 T	3.0 Tesla (magnetic field strength)
CT	Computed tomography
DEPA	Dose, Efficiency, Purity, Activation (PRP classification system)
FDG (in 18F-FDG)	Fluorodeoxyglucose
GLRLM	Grey-level run-length matrix
L-PRF	Leukocyte- and platelet-rich fibrin
L-PRP	Leukocyte- and platelet-rich plasma
MARSPILL	Method, Activation, Red blood cells, Spin, Platelets, Image guidance, Leukocytes, Light activation (PRP classification system)
MRI	Magnetic resonance imaging
MSK	Musculoskeletal
P-PRF	Pure platelet-rich fibrin
P-PRP	Pure platelet-rich plasma
PAW	Platelets, Activation, White cells (PRP classification system)
PET/CT	Positron emission tomography/computed tomography
PRP	Platelet-rich plasma
PRTEE	Patient-Rated Tennis Elbow Evaluation
PS-OCT	Polarisation-sensitive optical coherence tomography
SPADI	Shoulder Pain and Disability Index
T1ρ	Spin–lattice relaxation time in the rotating frame
T2	Transverse relaxation time
T2*	Effective transverse relaxation time
